# Probabilistic mapping of thalamic nuclei and thalamocortical functional connectivity in idiopathic generalised epilepsy

**DOI:** 10.1002/hbm.25644

**Published:** 2021-08-25

**Authors:** Yachin Chen, Nicholas Fallon, Barbara A. K. Kreilkamp, Christine Denby, Martyn Bracewell, Kumar Das, Emily Pegg, Rajiv Mohanraj, Anthony G. Marson, Simon S. Keller

**Affiliations:** ^1^ Department of Pharmacology and Therapeutics Institute of Systems, Molecular and Integrative Biology, University of Liverpool Liverpool UK; ^2^ The Walton Centre NHS Foundation Trust Liverpool UK; ^3^ Department of Psychology University of Liverpool Liverpool UK; ^4^ Department of Neurology University Medicine Göttingen Göttingen Germany; ^5^ Schools of Medical Sciences and Psychology, Bangor University Bangor UK; ^6^ Department of Neurology Manchester Centre for Clinical Neurosciences, Salford Royal NHS Foundation Trust Salford UK; ^7^ Division of Neuroscience and Experimental Psychology, Faculty of Biology, Medicine and Health School of Biological Sciences, University of Manchester Manchester UK

**Keywords:** epilepsy, functional MRI, pharmacoresistance, thalamus

## Abstract

It is well established that abnormal thalamocortical systems play an important role in the generation and maintenance of primary generalised seizures. However, it is currently unknown which thalamic nuclei and how nuclear‐specific thalamocortical functional connectivity are differentially impacted in patients with medically refractory and non‐refractory idiopathic generalised epilepsy (IGE). In the present study, we performed structural and resting‐state functional magnetic resonance imaging (MRI) in patients with refractory and non‐refractory IGE, segmented the thalamus into constituent nuclear regions using a probabilistic MRI segmentation method and determined thalamocortical functional connectivity using seed‐to‐voxel connectivity analyses. We report significant volume reduction of the left and right anterior thalamic nuclei only in patients with refractory IGE. Compared to healthy controls, patients with refractory and non‐refractory IGE had significant alterations of functional connectivity between the centromedian nucleus and cortex, but only patients with refractory IGE had altered cortical connectivity with the ventral lateral nuclear group. Patients with refractory IGE had significantly increased functional connectivity between the left and right ventral lateral posterior nuclei and cortical regions compared to patients with non‐refractory IGE. Cortical effects were predominantly located in the frontal lobe. Atrophy of the anterior thalamic nuclei and resting‐state functional hyperconnectivity between ventral lateral nuclei and cerebral cortex may be imaging markers of pharmacoresistance in patients with IGE. These structural and functional abnormalities fit well with the known importance of thalamocortical systems in the generation and maintenance of primary generalised seizures, and the increasing recognition of the importance of limbic pathways in IGE.

## Graphical abstract

We report that patients with idiopathic generalised epilepsy have different patterns of thalamic structural and functional connectivity alterations depending on whether they are refractory or not to anti‐seizure medication. Only refractory patients showed evidence of atrophy of the anterior (limbic) thalamic nuclei and increased functional connectivity between the ventral lateral posterior nuclei and cortex bilaterally.

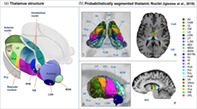

## 1 INTRODUCTION

Idiopathic generalised epilepsy (IGE) accounts for ~30% of all epilepsies (Behr, Goltzene, Kosmalski, Hirsch, & Ryvlin, 2016; Jallon & Latour, 2005). Approximately, 70% of all patients with IGE will be rendered seizure free after treatment with anti‐seizure medication (ASM; Alexopoulos, 2013; Faught, 2004). The pathophysiology of IGE has been associated with centrencephalic, corticoreticular and thalamocortical mechanisms (Avoli, 2012; Meeren, Van Luijtelaar, Lopes Da Silva, & Coenen, 2005). Spike–wave discharges (SWDs) in absence seizures have been attributed to thalamic dysfunction (Bal, von Krosigk, & McCormick, 1995; Hosford et al., 1995; Hosford et al., 1992; Huguenard & Prince, 1994; Liu, Vergnes, Depaulis, & Marescaux, 1992; McCormick & Bal, 1997; McCormick & Contreras, 2002; Prince & Farrell, 1969). This notion began to be established with Penfield and Jasper's centrencephalic theory, which suggested that the intralaminar nuclei were mainly associated with 3 Hz generalised SWDs via stimulation of this particular region of the thalamus (Meeren et al., 2005; Penfield & Jasper, 1954). The theory was later revised to suggest corticoreticular mechanisms for the generation of SWDs (Gloor, 1968). Rodent models have provided evidence for thalamic reticular mechanisms generating normal spindles and SWDs when thalamocortical systems are interrupted (Bal et al., 1995; McCormick & Bal, 1997; McCormick & Contreras, 2002). Studies suggested that a likely pathophysiology of SWDs was an aberrant GABAergic excitatory‐inhibitory modulation, majorly involving thalamic reticular cells and thalamocortical relay cells (von Krosigk, Bal, & McCormick, 1993). The cortical participation of extensive SWDs spread was further emphasised in Meeren's epileptic WAG/Rij rats study (Meeren, Pijn, Van Luijtelaar, Coenen, & Lopes da Silva, 2002). The wide synchronisation of SWDs was, therefore, thought to result from disturbances in thalamocortical networks, leading to continuous seizure presentation in IGE. Moreover, pathophysiological progression of IGE may differ in different thalamic nuclei (Norden & Blumenfeld, 2002; Wang, Zhang, Jiao, Liao, & Chen, 2012). Centromedian and anterior nuclei of the thalamus have been associated with initiation, propagation and maintenance of generalised SWDs (Miller & Ferrendelli, 1990; Miller, Hall, Holland, & Ferrendelli, 1989; Tyvaert et al., 2009). Although abnormal thalamocortical networks are clearly important in generating and maintaining generalised seizures, it is currently unknown whether differences in thalamocortical architecture and connectivity underlie pharmacoresistance in patients with IGE.

There have been many MRI‐based investigations of thalamic alterations in patients with IGE. Frequently used research methods include thalamic volume, thalamic shape and voxel‐based morphometry (VBM) analyses. While volume analysis attempts to evaluate gross thalamus volume change, shape and VBM analyses have allowed investigation of regional or subtle changes in the thalamus. Studies have reported a decrease (Boss, Abela, Weisstanner, Schindler, & Wiest, 2019; Ciumas & Savic, 2006; Du et al., 2011; Kim et al., 2014; Kim, Kim, Suh, & Kim, 2018; Lee, Seo, Lee, Kim, & Park, 2020; Mory et al., 2011; Nuyts, D'Souza, Bowden, & Vogrin, 2017; Perani et al., 2018; Pulsipher et al., 2009; Saini et al., 2013; Di Wang et al., 2016; Wang et al., 2012; Whelan et al., 2018; Zhong et al., 2018) and an increase (Betting et al., 2006; Bin et al., 2017; Lee, Seo, Lee, et al., 2020) in thalamic volume in patients with IGE compared to healthy controls. Some studies have, however, reported no statistically significant differences in thalamic volume (Betting et al., 2010; Natsume, Bernasconi, Andermann, & Bernasconi, 2003; Seeck et al., 2005). This discrepancy may be due to a number of factors, including that thalamic nuclei are potentially differentially impacted in IGE, differential combinations of refractory and / or non‐refractory patients in statistical analyses, and different analytical approaches were adopted. Kim and colleagues reported grey matter (GM) atrophy in the anteromedial thalamus in IGE relative to controls (Kim et al., 2014). Two other studies that specifically focused on juvenile myoclonic epilepsy (JME) suggested volume reduced in the anterior (Mory et al., 2011) and medial thalamus (Saini et al., 2013) in patients relative to controls. Wang and colleagues also identified thalamic atrophy in the medial dorsal and pulvinar nuclei in patients with IGE generalised tonic–clonic seizure alone (IGE‐GTCS) relative to controls (Wang et al., 2012). Localised volume reduction of the ventral thalamus has been reported in patients with refractory IGE relative to controls (Boss et al., 2019). There is therefore accumulating evidence indicating regionally specific structural alterations of the thalamus in IGE, but there is a lack of consistency with respect to the particular region of the thalamus affected.

There have been many approaches to segment thalamic nuclei using MRI; many of these necessitate the use of high field or advanced MRI sequences that may not be incorporated to clinical scanning for patients with epilepsy. A recently published thalamic nuclei segmentation method provides an opportunity to probabilistically segment the thalamus into 50 thalamic nuclei based on histological data and ex‐vivo MRI and necessitates only three dimensional T1‐weighted (T1w) MRI data (Iglesias et al., 2018). The approach has been recently applied to determine regionally specific thalamic alterations in neurological disorders (Bocchetta et al., 2020; Hougaard et al., 2020; Ngamsombat et al., 2020; Shin, Lee, & Park, 2019) but has not yet been applied to patients with IGE. In the present study, we use this method to determine regional structural and functional thalamic abnormalities in patients with IGE and differences according to seizure control.

Previous simultaneous EEG‐fMRI studies have reported that the thalamus is activated during generalised SWDs (Centeno & Carmichael, 2014; Klamer et al., 2018; Seneviratne, Cook, & D'Souza, 2014; Tyvaert et al., 2009). Large‐scale network analysis using resting‐state functional MRI (rs‐fMRI) has revealed altered functional connectivity in patients with IGE relative to controls, which were often found in intrinsic networks, such as the default mode and salience networks (Li et al., 2017; McGill et al., 2012; Wang et al., 2011; Wei et al., 2015). Few studies have identified thalamocortical functional alterations in IGE using rs‐fMRI. Serving as a functional complex and relay station between different subcortical areas and the cerebral cortex, distinct thalamic nuclei may be divergently associated with functionally distinct areas in cortex (Mai & Majtanik, 2019; Noback, Strominger, Demarest, & Ruggiero, 2005; Rikhye, Wimmer, & Halassa, 2018; Yuan et al., 2016). This suggests that thalamocortical functional connectivity alterations in IGE might also be divergent depending on the involvement of particular thalamic nuclei—potentially differing between patients with refractory and non‐refractory IGE. Some studies have reported reduced *or* increased thalamocortical rs‐fMRI connectivity when the regional thalamic seed was generated based on VBM results (Kim et al., 2014; Wang et al., 2012; Zhong et al., 2018) or diffusion tensor imaging connectivity‐based thalamic parcellations (Jiang et al., 2018). However, there has been no work administered to date that has explored the functional connectivity between cortex and the delineated nuclei of the thalamus, while investigating potential differences in thalamocortical connectivity in patients with refractory and non‐refractory IGE.

There were two primary goals of the present study. First, we sought to determine regional thalamic nuclear volume alterations in patients with refractory and non‐refractory IGE and healthy controls. We hypothesised differential thalamic nuclei involvement based on whether patients were refractory or non‐refractory to ASM. Second, using parcellated thalamic nuclei regions as seeds, we sought to investigate rs‐fMRI thalamocortical connectivity in IGE and to determine whether alterations are associated with treatment outcomes. To our knowledge, this represents the first study to probe differences in thalamocortical architecture and connectivity in patients with refractory and non‐refractory IGE.

## 2 METHODS

### 2.1 Participants

Thirty‐five patients with a diagnosis of IGE were prospectively recruited at the Walton Centre NHS Foundation Trust (Liverpool, United Kingdom) and Salford Royal NHS Foundation Trust (Manchester, United Kingdom). All patients had a diagnosis of childhood absence epilepsy, juvenile absence epilepsy, juvenile myoclonic epilepsy or epilepsy with generalised tonic–clonic seizures alone. Based on the ILAE clinical guidelines (Fisher et al., 2017; Scheffer et al., 2017). Patients were prospectively recruited based on their seizure control; those who experienced two or more seizures in the past 12 months were classified as medically refractory and patients who had been seizure‐free in the preceding 12 months were classified as medically non‐refractory. Seizure frequency and 12 months seizure freedom were determined by self‐report from patients, their family and witnesses and regular clinical evaluation. Exclusion criteria included patients with either progressive neurological disease or confirmed focal abnormality indicated in clinical MRI. A breakdown of the patients' clinical information is provided in Table 1. We also recruited 39 healthy volunteers with no history of neurological or psychiatric illness to serve as a control cohort. Comparison between patient and control demographic data is presented in Table 2. There were no differences in sex between patients and controls, but a difference in age between patients with non‐refractory IGE and controls.

**TABLE 1:** Clinical characteristics of patients. Year as the unit for age, onset and duration

Patient	Age	Sex	Onset	Duration	Category	FH	PS	Seizures	ASM (mg/day)
1	34	F	2	32	REF	N	N	GTCS	VPA 3000, ZON 300
2	23	F	14	9	REF	N	N	AS, MS	LEV 300, TOP 300, Clob 10
3	19	M	16	3	REF	Y	Y	GTCS	VPA 1000
4	19	F	8	11	REF	Y	N	AS, GTCS	LTG 200
5	25	M	19	6	nonREF	N	N	MS	LEV1500, VPA 1600
6	60	F	13	47	REF	Y	N	AS, GTCS	VPA 2500
7	24	M	15	9	REF	Y	N	AS, MS, GTCS	CBZ1000, LEV 3000, VPA 2500
8	21	F	15	6	REF	N	N	AS, MS, GTCS	LEV 4000, VPA 2000
9	32	F	23	9	REF	Y	N	MS, GTCS	LEV 3500, Clob 15
10	38	M	18	20	REF	Y	N	GTCS	VPA 600, LTG 50
11	67	M	29	38	REF	N	N	AS, GTCS	VPA 2000, LTG 200, PB 150, Clob 10
12	46	F	7	39	REF	N	N	AS	VPA 1200, LTG 200, LEV 2500
13	20	M	8	12	REF	N	N	GTCS	VPA 2000
14	24	F	13	11	REF	Y	N	MS, GTCS	TOP 100
15	35	M	6	29	REF	N	N	GTCS	LEV 2000, VPA 2000
16	18	M	14	4	REF	N	N	AS, GTCS	VPA 1500, ZON 350
17	39	M	17	22	REF	Y	Y	GTCS	VPA 1000, LTG 75
18	24	F	16	8	nonREF	N	N	AS, GTCS	VPA 1000, LTG 200, LEV 4000
19	21	M	16	5	REF	N	N	AS, MS, GTCS	VPA 2400
20	36	F	17	19	REF	N	N	GTCS	LEV 1250, TOP 100
21	31	F	15	16	REF	N	N	GTCS	LEV 2000, LTG 400
22	31	F	16	15	REF	N	N	AS, MS, GTCS	VPA 1500, LEV 3500
23	23	M	16	7	nonREF	N	N	AS, GTCS	VPA 2100, LEV 500
24	19	F	13	6	nonREF	Y	N	GTCS	LEV 3000
25	58	F	15	43	REF	N	N	GTCS	VPA 1000, ZON 400, Clon 1.5
26	18	F	15	3	nonREF	N	Y	AS, MS	LEV 2000
27	22	M	2	20	nonREF	N	Y	AS, MS	VPA 1400
28	24	M	13	11	REF	N	N	MS	VPA 1700
29	56	F	3	53	nonREF	N	Y	AS	VPA 1500
30	57	F	7	50	REF	N	N	AS, GTCS	VPA 1200, CBZ 600
31	33	M	7	26	nonREF	N	N	AS	VPA 1800
32	19	F	14	5	nonREF	N	N	AS, MS	LEV 1000
33	57	F	7	50	REF	Y	N	GTCS	VPA 2000, LTG 75
34	20	M	16	4	nonREF	N	N	AS, GTCS, MS	VPA 1700, ETX 500

*Note*: Onset indicates the age of onset of epilepsy.

Abbreviations: AS, absence seizures; ASM, anti‐seizure medication (daily dose in milligramme); Clob, clobazam; Clon, clonazepam; CBZ, carbamazepine; ETX, ethosuximide; F, female; FH, family history; GTCS, primary generalised tonic–clonic seizures; LEV, Levetiracetam; LTG, lamotrigine; M, male; MS, myoclonic seizures; N, no; PB, phenobarbital; PS, photosensitive; REF, refractory; TOP, topiramate; VPA, Valproic acid; Y, yes; ZON, zonisamide.

**TABLE 2:** Demographics for healthy controls, patients and subgroup of patients according to treatment response

	Group			Stats
	Controls	nonREF‐IGE	REF‐IGE	
*N*	39	10	24	
Age (*SD*), range	32.1 (8.6), 21–60	25.9 (11.4), 18–56	34.8 (15.1), 18–67	χ^2^(2,72) = 6.99, *p* = .03
Sex (F/M)	23/16	5/5	14/10	χ^2^(2) = 0.27, *p* = .87
Mean age of onset (*SD*)	N/A	12.1 (5.9)	13.5 (5.8)	*Z* = −0.32, *p* = .74
Mean duration corrected for age (*SD*)	N/A	0.4 (0.3)	0.6 (0.2)	*Z* = −1.15, *p* = .24

*Note*: Chi‐squared test of independence was conducted for sex variables. Kruskal–Wallis ANOVA was conducted for age differences between three groups. Wilcoxon‐Rank‐Sum test was used for age of onset of epilepsy and duration of epilepsy corrected for age.

Abbreviations: F, female; IGE, idiopathic generalised epilepsy; M, male; REF, refractory.

### 2.2 MRI acquisition

MRI acquisition for all participants was performed at the Department of Neuroradiology, Walton Centre NHS Foundation Trust using a 3.0 Tesla General Electric Discovery MR750 scanner with a 32‐channel head coil. Sequences used for analysis in this paper included: (a) 3D axial T1w fast spoiled gradient echo (FSPGR) MRI with Phased Array Uniformity Enhancement (PURE) signal inhomogeneity correction (pulse sequence = BRAVO; TR = 8.2 ms, TI = 450 ms, TE = 3.22 ms, flip angle = 12, slice thickness = 1 mm, voxel size = 0.94 mm × 0.94 mm, 136 slices, FOV = 24 cm) and (b) resting‐state functional MRI using a gradient echo EPI sequence (TR = 2000 ms, TE = 25 ms, flip angle = 75, slice thickness = 2.4 mm, voxel size = 3.75 mm × 3.75 mm, 180 volumes, 38 slices, FOV = 24 cm). No task was used, and sedation was not administered during scanning. For all participants, image acquisition was performed while awake and with visual fixation on a white crosshair with a black background.

### 2.3 Analysis of thalamic nuclei volumes

**FIGURE 1:**
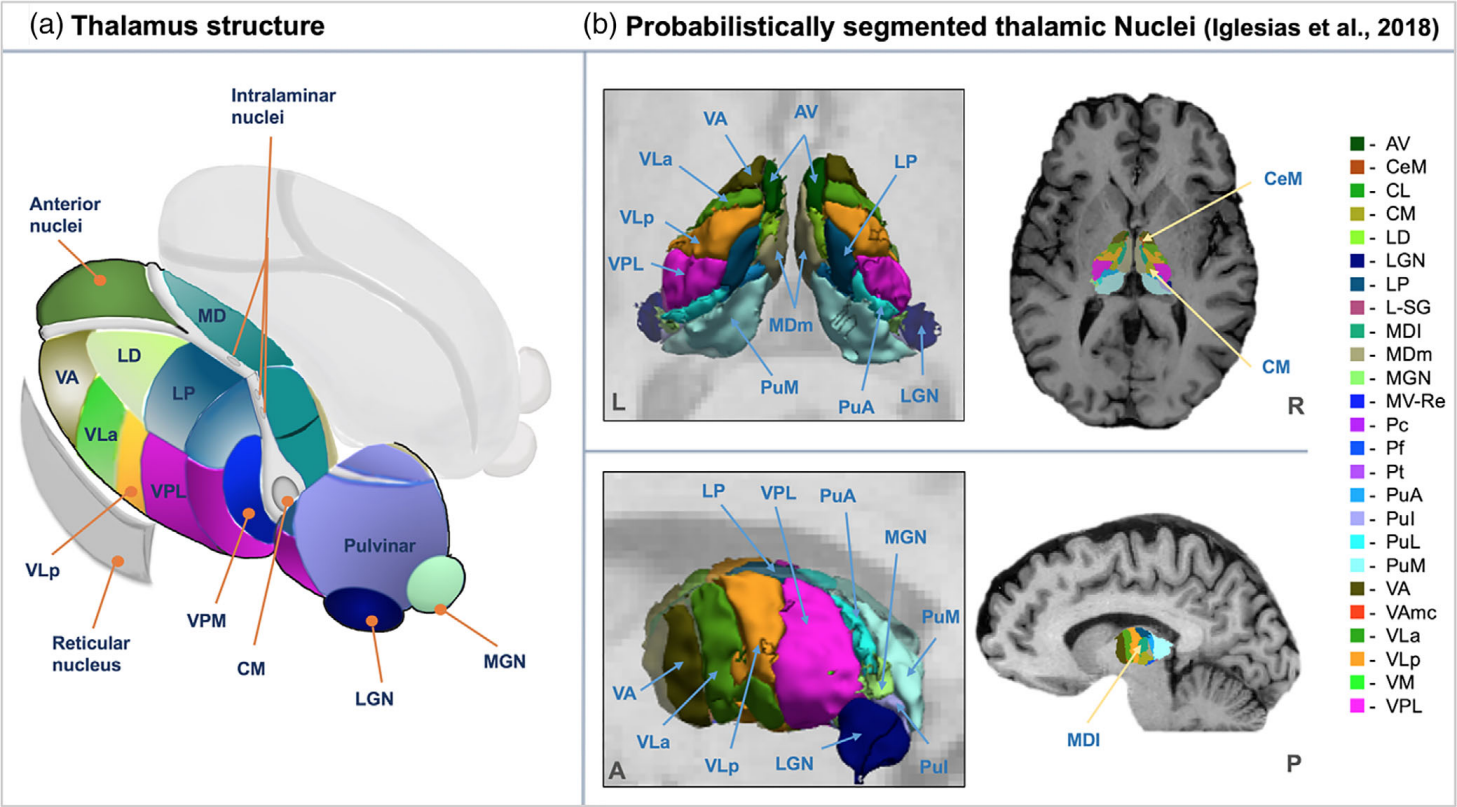
Thalamic nuclei. (a) Schematic illustration of the anatomical location of thalamic nuclei. (b) Probabilistic segmentation of thalamus using FreeSurfer (Iglesias et al., 2018). MRI was taken from one control subject in the present study cohort

FreeSurfer (http://surfer.nmr.mgh.harvard.edu, version 6.0, Fischl, 2012) and a probabilistic thalamic segmentation algorithm incorporated into FreeSurfer software were applied to segment and estimate the volumes of thalamic nuclei. First, T1w images were processed with the FreeSurfer “recon‐all” function to correct for non‐uniformity and fluctuations in MRI intensity, remove skull, and perform an automated intensity‐based segmentation for cortical and subcortical brain structures. The second step was to segment the left and right thalamus each into 25 different nuclei using Bayesian inference based on a probabilistic atlas built with histological data (Iglesias et al., 2018). The parcellated thalamic nuclei are illustrated in Figure 1; abbreviations for each nucleus are presented in Table 3. We divided the nuclear volume measurements by the respective total intracranial volume estimated using FreeSurfer (Buckner et al., 2004) in order to control for the effect of brain size. The following formula was used:

$$\text{Nuclei volume}\;\left(\%\right)=\left(\text{nuclei volume}\;\left({\mathrm{mm}}^{3}\right)/\text{total intracranial volume}\;\left({\mathrm{mm}}^{3}\right)\right)\times 100\%$$

**TABLE 3:** Thalamic nuclei segmented in the present study. Grouping was based on Iglesias's et al. (2018)

Group	Abbreviation	Nucleus
Anterior	AV	Anteroventral
Ventral	VA	Ventral anterior
	VAmc	Ventral anterior magnocellular
	VLa	Ventral lateral anterior
	VLp	Ventral lateral posterior
	VM	Ventromedial
	VPL	Ventral posterolateral
Medial	MDl	Mediodorsal lateral parvocellular
	MDm	Mediodorsal medial magnocellular
	MV‐Re	Reuniens (medial ventral)
	Pt	Paratenial
Lateral	LD	Laterodorsal
	LP	Lateral posterior
Posterior	PuA	Pulvinar anterior
	PuM	Pulvinar medial
	PuI	Pulvinar inferior
	PuL	Pulvinar lateral
	LGN	Lateral geniculate
	MGN	Medial geniculate
	L‐SG	Limitans (suprageniculate)
Intralaminar	CeM	Central medial
	CM	Centromedian
	CL	Central lateral
	Pc	Paracentral
	Pf	Parafascicular

Analysis was performed using a one‐way analysis of covariance (ANCOVA) in SPSS 25 (IBM Corporation, Armonk, NY), where each nuclear volume was compared among all three groups (controls, patients with refractory IGE and those with non‐refractory IGE) while controlling for effects of age and sex. A false discovery rate (FDR) approach (Benjamini & Hochberg, 1995) was applied to correct for multiple comparisons using MATLAB 2018a (The Mathworks, Inc., 2018) Bioinformatics Toolbox. An FDR‐adjusted *p*‐value of less than .05 was considered statistically significant.

### 2.4 Thalamic functional connectivity

As a first step, all imaging data were manually recentered to the anterior commissure. Rs‐fMRI data were preprocessed using Statistical Parametric Mapping software (SPM12, Welcome Trust Centre for Neuroimaging, London, United Kingdom; https://www.fil.ion.ucl.ac.uk/spm/) and the Computational Anatomy Toolbox (CAT12; http://www.neuro.uni-jena.de/cat/) in MATLAB 2018a. Functional image preprocessing included slice‐timing correction, motion estimation, spatial normalisation to MNI space and spatial smoothing. First, rs‐fMRI slice timing was corrected using the first image volume as the reference. Second, head motion parameters and movement‐by‐susceptibility induced variance in the fMRI EPI sequence were estimated using the realign and unwarp functions. The movement parameters were further included as covariance in the general linear model for functional connectivity analysis. Quality control for motion artefacts was performed by employing a threshold (translation > 3 mm, rotation > 1°) for exclusion (Fallon, Chiu, Nurmikko, & Stancak, 2016; Johnstone et al., 2006). Functional data were subsequently spatially normalised to MNI space using the ICBM 152 template of European brains and interpolated to isotropic 2 mm voxels using the fourth degree B‐Spline method. The mean functional image generated from the previous step for each subject was initially selected as the target for image registration. All images were smoothed using an 8 mm full‐width half‐maximum (FWHM) Gaussian kernel at every data point. T1w images additionally underwent automated tissue segmentation into grey/white matter and cerebrospinal fluid (CSF) compartments using CAT12 followed by a non‐linear registration to the ICBM 152 template. MNI registrations for all image data and segmentations were visually inspected to confirm accuracy and each individual's thalamic nuclear mask was checked for alignment with MNI thalamic masks provided in CONN.

**FIGURE 2:**
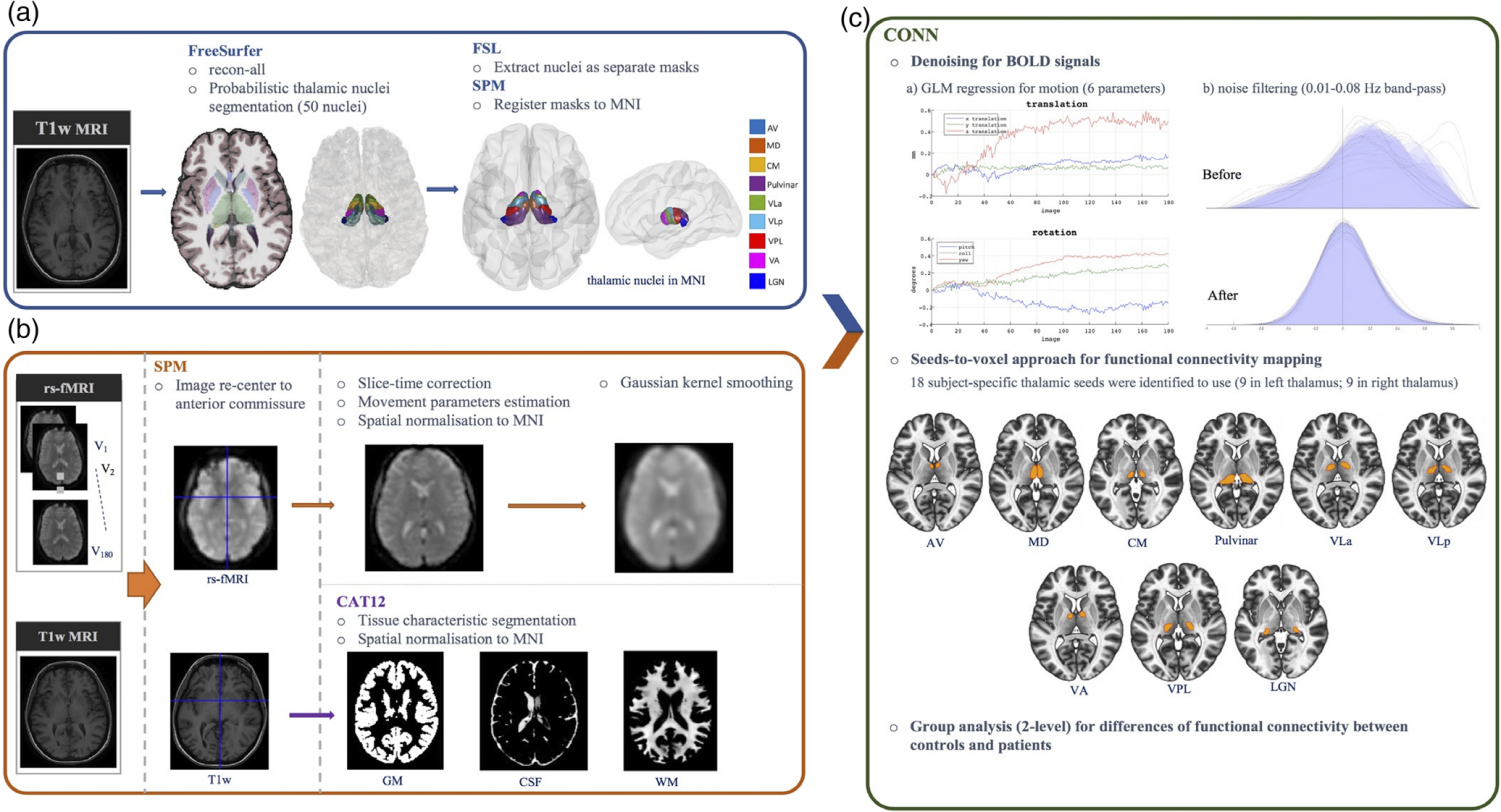
A framework for resting‐state thalamocortical functional connectivity analysis. (a) Identifying thalamic regions of interest as per Figure 1. All nuclei were extracted and binarized into 50 separate masks using FSL. Nuclei selected for analysis were registered to ICBM 152 template. (b) rs‐fMRI pre‐processing based on the standard SPM protocol. The origin of T1w and rs‐fMRI data was set to the anterior commissure prior to all processing. rs‐fMRI data underwent corrections, normalisation and spatial smoothing; T1w data underwent tissue characteristic segmentation and spatial normalisation. (c) CONN processing for resting state functional connectivity. All pre‐processing data (from a and b) were input into CONN toolbox for running functional connectivity analysis. The seed‐to‐voxel approach was used. Movement parameters were regressed, and noise was filtered to produce clean BOLD signals. Second‐level analysis was implemented to determine significant differences of rs‐fMRI thalamocortical connectivity between study groups

A methods workflow of rs‐fMRI thalamocortical analysis is provided in Figure 2. Resting‐state functional connectivity analysis was performed using the Functional Connectivity Toolbox (CONN; https://web.conn-toolbox.org, Whitfield‐Gabrieli & Nieto‐Castanon, 2012) integrated in SPM12. The toolbox implemented two general steps of BOLD signal denoising: (1) linear regression to remove effects of confounding variables and (2) band‐pass filtering for extraneous and physiological noise. Noise components from WM and CSF were removed based on principal component analysis of the multivariate BOLD signal within GM masks produced from T1w tissue segmentation for each subject. This removal was specified as an anatomical component‐based noise correction procedure (Behzadi, Restom, Liau, & Liu, 2007). Furthermore, each subject's motion parameters were included as regressors (Figure 2c, denoising [a]). A conventional band‐pass filter at 0.01 Hz–0.08 Hz frequency band was employed to minimise scanner drift, vascular and physiological fluctuations (Boubela et al., 2013; Davey, Grayden, Egan, & Johnston, 2013; Fox & Raichle, 2007; Yuen, Osachoff, & Chen, 2019). For analysis of functional connectivity, we explored group differences in thalamocortical connectivity through the analysis of statistical dependencies of functional data between thalamic and other cortical and subcortical GM regions of interest using the seed‐to‐voxel approach. We selected thalamic nuclei which relay information to specific cortical areas as the seeds of interest, including anteroventral (AV), ventral lateral anterior (VLa), ventral lateral posterior (VLp), ventral anterior (VA), and lateral geniculate nucleus (LGN) (Table 3). Moreover, centromedian (CM), mediodorsal (MD) and pulvinar regions were also included for analysis due to their association with epilepsy (Avoli, Gloor, Kostopoulos, & Gotman, 1983; Inoue, Duysens, Vossen, & Coenen, 1993; Ji et al., 2015; Kato et al., 2008; Miller et al., 1989; Tyvaert et al., 2009; Valentín et al., 2013; Wang et al., 2012). The MD seed was generated by combining MDl and MDm (Table 3) due to shared functional topology and the small size of those seeds (Mitchell & Chakraborty, 2013; Pergola et al., 2018). The pulvinar seed was defined as a collection of PuA, PuM, PuI and PuL (Table 3). All nuclei were then spatially normalised to MNI space using SPM tools.

To compute seed‐based functional connectivity, the resting‐state BOLD time series for each seed ROI was averaged and correlated with BOLD time series for each GM voxel. Fisher‐transformed bivariate correlation coefficients were calculated to represent the degree of functional connectivity. The seed‐based connectivity maps were subsequently used for the second‐level analysis of relative thalamocortical functional connectivity changes between groups. A statistical parametric map was created to characterise the differences in functional connectivity between groups using the general linear model, corrected for age and sex. Voxel‐wise statistics for thalamocortical connectivity throughout the entire brain were controlled at an uncorrected level (*p* < .001) with an additional cluster‐level correction (*p*_*FDR*_ < .05) based on Gaussian Random Field theory (Worsley et al., 1996) applied for FDR correction (Alonazi et al., 2019; Chumbley, Worsley, Flandin, & Friston, 2010; Fallon et al., 2016). Identification of anatomical regions for significant clusters was based on Harvard‐Oxford atlas (Makris et al., 2006).

## 3 RESULTS

### 3.1 Thalamic nuclei volumetric analysis

**FIGURE 3:**
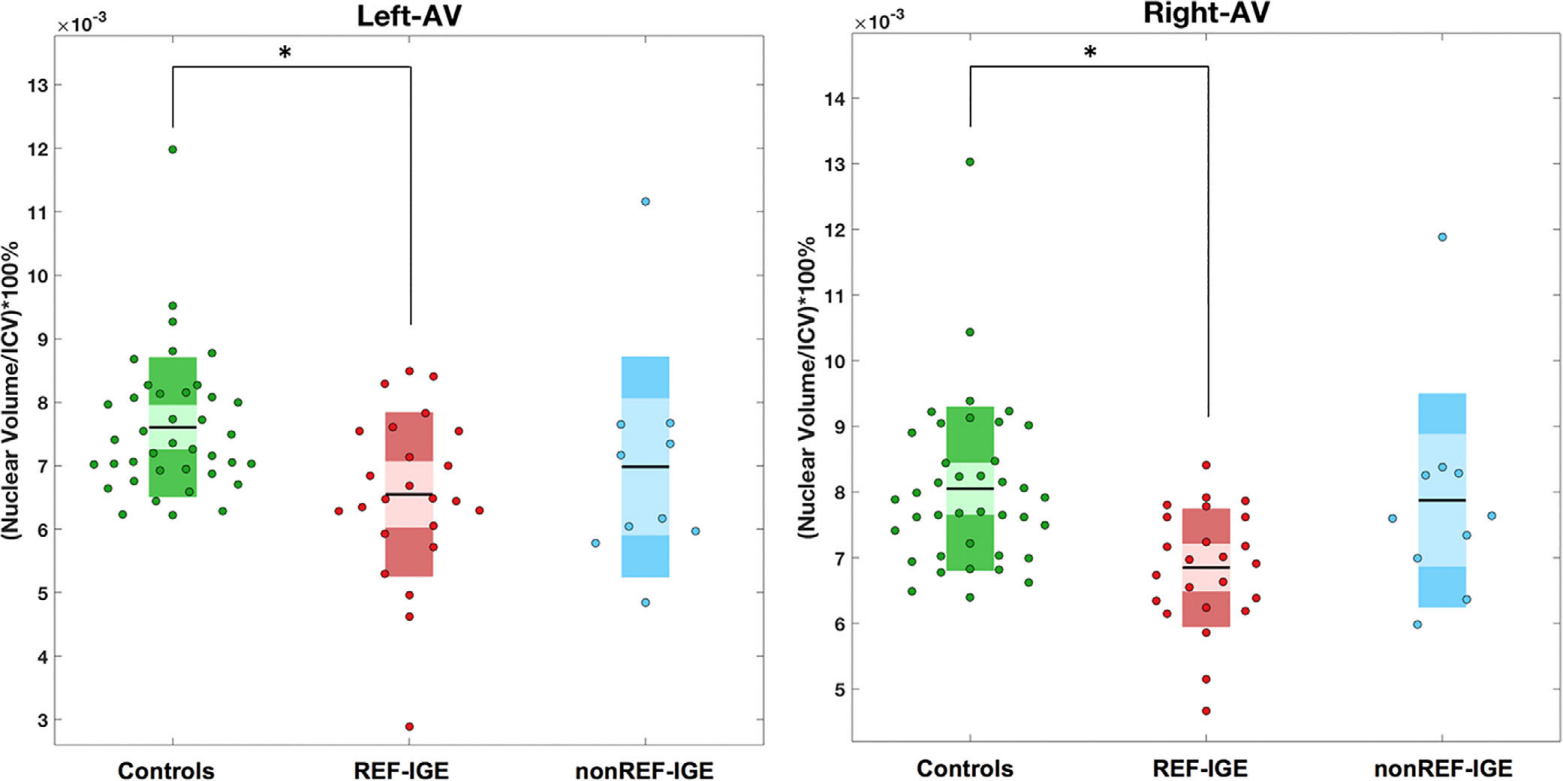
Significant volume reduction of the left and right anteroventral (AV) only in patients with refractory idiopathic generalised epilepsy (IGE) as revealed by one‐way analysis of covariance (ANCOVA). Asterisks indicate significant differences between groups at *p*_FDR_

Statistically significant volume reduction was only observed in patients with refractory IGE compared to healthy controls (left AV, *p*_*FDR*_ = .025; right AV, *p*_*FDR*_ = .018) (Figure 3). There was no trend for volume reduction of the left or right AV in patients with non‐refractory IGE compared to controls (Table 4). Trends were observed for volume reduction of other thalamic nuclei in patients compared to controls, but these effects did not survive FDR correction (Table 4).

**TABLE 4:** Thalamic nuclear volume and group differences

Thalamic nucleus	Volume corrected for ICV, ratio (10^−3^)				Stats				
	Control	IGE	REF‐IGE	nonREF‐IGE	Control versus IGE	Control versus REF‐IGE	Control versus nonREF‐IGE	nonREF‐IGE versus REF‐IGE
	Mean (*SD*)	Mean (*SD*)	Mean (*SD*)	Mean (*SD*)	*p*_*uncorr*_	*p*_*uncorr*_	*p*_*uncorr*_	*p*_*uncorr*_
L‐MGN	6.59 (1.13)	6.74 (1.19)	6.53 (1.02)	7.24 (1.47)	.63	.97	.23	.24
L‐LGN	13.54 (1.52)	13.53 (1.99)	12.85 (1.33)	15.15 (2.42)	.97	.12	.03^a^	.003^a^
L‐PuI	14.71 (2.20)	14.95 (2.73)	14.26 (1.93)	16.60 (3.69)	.70	.47	.11	.06
L‐PuM	72.24 (7.23)	74.46 (11.27)	71.55 (7.64)	81.4 (15.51)	.35	.79	.04^a^	.06
L‐LSg	1.15 (0.28)	1.21 (0.28)	1.18 (0.27)	1.27 (0.29)	.46	.61	.38	.78
L‐VPL	52.43 (5.37)	51.19 (6.93)	49.45 (4.12)	55.36 (10.26)	.32	.03^a^	.35	.05^a^
L‐CM	15.81 (1.66)	15.68 (2.01)	15.19 (1.60)	16.85 (2.47)	.70	.18	.27	.09
L‐VLa	39.20 (3.79)	38.57 (4.98)	37.44 (2.99)	41.28 (7.52)	.43	.07	.42	.12
L‐PuA	15.26 (1.39)	15.35 (2.10)	14.91 (1.38)	16.38 (3.09)	.90	.34	.17	.16
L‐MDm	52.46 (4.88)	52.67 (7.77)	50.79 (5.53)	57.19 (10.54)	.95	.22	.12	.08
L‐Pf	3.63 (0.33)	3.53 (0.46)	3.43 (0.40)	3.77 (0.53)	.18	.03^a^	.48	.12
L‐VAmc	2.12 (0.24)	2.08 (0.26)	2.04 (0.22)	2.18 (0.32)	.44	.19	.74	.36
L‐MDl	18.93 (1.85)	18.61 (2.72)	18.10 (2.06)	19.85 (3.72)	.38	.09	.46	.24
L‐CeM	4.31 (0.61)	3.98 (0.80)	4.01 (0.77)	3.91 (0.90)	.03^a^	.07	.11	.32
L‐VA	25.94 (2.56)	25.99 (3.20)	25.52 (2.42)	27.11 (4.53)	1.00	.63	.43	.44
L‐MV(Re)	0.84 (0.13)	0.766 (0.19)	0.76 (0.18)	0.77 (0.22)	.03^a^	.04^a^	.21	.61
L‐VM	1.17 (0.13)	1.12 (0.14)	1.08 (0.11)	1.20 (0.19)	.10	.01^a^	.74	.07
L‐CL	1.85 (0.42)	1.60 (0.56)	1.56 (0.36)	1.70 (0.36)	.01^a^	.01^a^	.31	.74
L‐PuL	11.79 (1.73)	11.95 (2.04)	11.68 (1.83)	12.59 (2.48)	.71	.88	.28	.43
L‐Pt	0.43 (0.05)	0.42 (0.06)	0.41 (0.04)	0.46 (0.08)	.33	.05^a^	.38	.09
L‐AV	7.61 (1.10)	6.68 (1.42)	6.55 (1.29)	6.98 (1.74)	.001^a^	.001^b^	.14	.95
L‐Pc	0.24 (0.03)	0.23 (0.04)	0.23 (0.04)	0.25 (0.04)	.66	.31	.45	.29
L‐VLp	50.83 (5.13)	49.59 (7.01)	47.88 (4.08)	53.69 (10.56)	.29	.02^a^	.44	.09
L‐LP	7.15 (1.82)	6.36 (1.90)	5.92 (1.53)	7.42 (2.36)	0.04^a^	.01^a^	.80	.13
R‐LGN	13.80 (1.82)	13.99 (1.90)	13.57 (1.24)	15.02 (2.77)	0.71	.67	.15	.09
R‐MGN	6.42 (0.95)	6.62 (1.66)	6.27 (1.00)	7.44 (2.54)	0.49	.84	.07	.17
R‐PuI	14.30 (2.23)	14.35 (2.41)	13.84 (2.05)	15.58 (2.87)	0.98	.49	.34	.11
R‐PuM	68.48 (6.25)	68.38 (10.48)	65.83 (7.22)	74.50 (14.51)	0.88	.18	.15	.08
R‐LSg	1.15 (0.27)	1.19 (0.35)	1.13 (0.28)	1.35 (0.46)	0.62	.79	.11	.16
R‐VPL	49.56 (4.41)	48.59 (5.89)	47.28 (4.07)	51.73 (8.33)	0.35	.05	.45	.10
R‐CM	15.31 (1.61)	14.98 (1.84)	14.59 (1.48)	15.91 (2.35)	0.38	.11	.59	.17
R‐VLa	39.44 (3.63)	38.84 (4.28)	37.99 (2.77)	40.89 (6.39)	0.40	.11	.55	.22
R‐PuA	14.82 (1.34)	14.78 (2.02)	14.33 (1.43)	15.84 (2.82)	0.81	.19	.20	.11
R‐MDm	51.66 (5.39)	50.53 (8.21)	48.59 (5.56)	55.18 (11.59)	0.32	.03^a^	.38	.08
R‐Pf	3.66 (0.35)	3.55 (0.50)	3.43 (0.41)	3.83 (0.60)	0.20	.01^a^	.36	.08
R‐VAmc	2.18 (0.19)	2.17 (0.21)	2.14 (0.18)	2.25 (0.24)	0.74	.39	.50	.37
R‐MDl	18.91 (2.20)	18.33 (2.73)	17.73 (1.88)	19.79 (3.88)	0.21	.03^a^	.62	.11
R‐VA	26.13 (2.29)	26.81 (2.84)	26.31 (2.11)	28.01 (3.99)	0.31	.70	.10	.31
R‐MV(Re)	0.87 (0.14)	0.78 (0.18)	0.78 (0.19)	0.79 (0.17)	0.02^a^	.03^a^	.14	.64
R‐CeM	4.56 (0.61)	4.26 (0.75)	4.27 (0.74)	4.25 (0.82)	0.05^a^	.07	.25	.67
R‐VM	1.11 (0.12)	1.07 (0.13)	1.05 (0.11)	1.11 (0.16)	0.08	.03^a^	.76	.33
R‐PuL	10.82 (1.64)	11.06 (1.92)	10.79 (1.65)	11.71 (2.44)	0.63	.99	.23	.32
R‐CL	1.92 (0.43)	1.65 (0.44)	1.57 (0.40)	1.85 (0.49)	0.02^a^	.01^a^	.92	.20
R‐VLp	50.65 (4.87)	48.84 (5.95)	47.51 (3.71)	52.03 (8.88)	0.10	.01^a^	.82	.14
R‐Pc	0.24 (0.03)	0.23 (0.03)	0.23 (0.03)	0.24 (0.03)	0.47	.40	1.00	.83
R‐Pt	0.41 (0.04)	0.39 (0.06)	0.38 (0.05)	0.42 (0.07)	0.21	.04^a^	.48	.10
R‐AV	8.05 (1.25)	7.15 (1.23)	6.85 (0.90)	7.87 (1.62)	0.004^a^	.0004^b^	.80	.09
R‐LP	6.65 (1.41)	5.95 (1.66)	5.64 (1.44)	6.71 (1.98)	0.06	.02^a^	.82	.29
L‐LD	1.49 (0.59)	1.18 (0.62)	1.11 (0.64)	1.33 (0.57)	0.02^a^	.02^a^	.48	.76
R‐LD	1.32 (0.49)	1.07 (0.61)	0.99 (0.63)	1.26 (0.57)	0.09	.05^a^	.88	.49
L‐Thal	421.71 (34.55)	418.40 (54.83)	404.41 (32.66)	451.95 (80.78)	0.64	.07	.19	.07
R‐Thal	412.41 (32.40)	405.57 (51.36)	393.09 (31.03)	435.54 (76.15)	0.39	.03^a^	.31	.08

Abbreviations: L, left side; R, right side; Thal, thalamus.

^a^ Uncorrected significance.

^b^ FDR corrected significance (*p*_*FDR*_ < .05).

### 3.2 Thalamocortical resting‐state functional connectivity analysis

**FIGURE 4:**
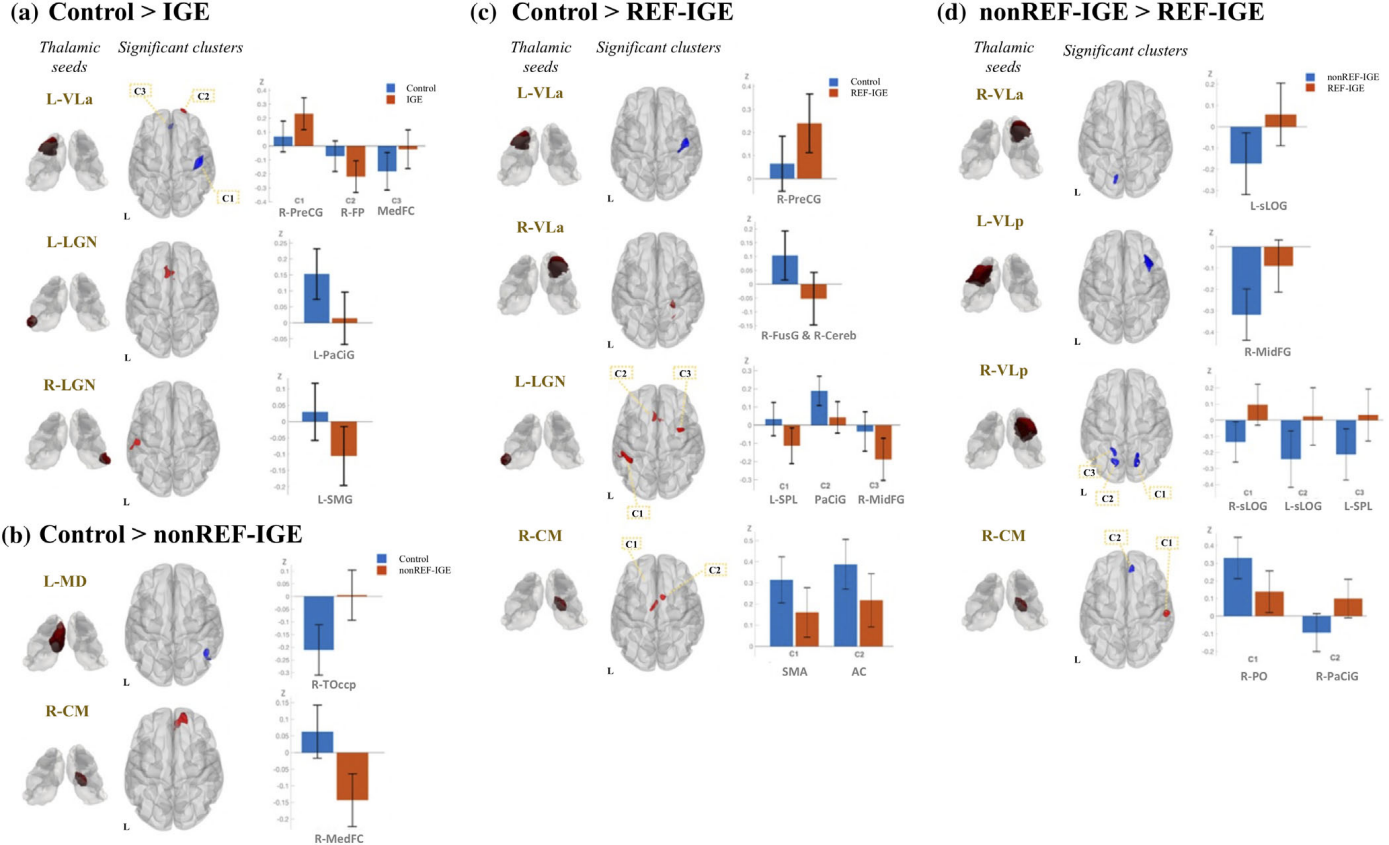
Significant differences in thalamocortical functional connectivity between groups. Boxplots of mean Fischer transformed correlation coefficients illustrate correlation (positive) and anti‐correlation (negative) relationship of individual thalamocortical functional connectivity. (a) Functional connectivity alteration with thalamic seeds in patients with IGE relative to controls. Cluster colour indicates the direction of contrast (red: control > IGE; blue: IGE > control); (b) Functional connectivity alteration with thalamic seeds in patients with non‐refractory IGE relative to controls. Cluster colour indicates the direction of contrast (red: Control > nonREF‐IGE; blue: nonREF‐IGE > Control); (c) Functional connectivity alteration with thalamic seeds in patients with refractory IGE relative to controls. Cluster colour indicates the direction of contrast (red: control > REF‐IGE; blue: REF‐IGE > Control); (d) Functional connectivity difference between patients with non‐refractory IGE and patients with non‐refractory IGE. Cluster colour indicates the direction of contrast (red: nonREF‐IGE > REF‐IGE; blue: REF‐IGE > nonREF‐IGE). AC, anterior cingulate gyrus; Cereb, cerebellum; FP, frontal pole; FusG, fusiform gyrus; L, left; MedFC, frontal medial cortex; MidFG, middle frontal gyrus; PaCiG, paracingulate gyrus; PO, parietal operculum cortex; PreCG, precentral gyrus; R, right; SMA, supplementary motor area; SMG, supramarginal gyrus; sLOG, lateral occipital gyrus (superior part); SPL, superior parietal lobule; TOccp, temporooccip

Patients with IGE had significantly altered functional connectivity between thalamic seeds—including left VLa and LGN bilaterally—and regions of the cerebral cortex, relative to controls (Figure 4a). Significantly increased thalamocortical connectivity was observed between left VLa and frontal medial cortex and right precentral gyrus in patients relative to controls. Conversely, significantly decreased thalamocortical connectivity was observed between left VLa and right frontal pole, left LGN and left paracingulate gyrus, and right LGN and left supramarginal gyrus in patients with IGE compared to controls (Table 5).

**TABLE 5:** Significant differences in functional thalamocortical connectivity between groups, based on seed‐to‐voxel analysis

Contrast	Seeds	MNI coordinate (mm)			*k*	|T|	*p*_*FDR*_	Cluster
		*X*	*Y*	*Z*				
Control > IGE	L‐VLa	20	70	‐4	106	4.74	.038	Right frontal pole
	L‐LGN	‐4	24	44	359	6.31	.00013	Left paracingulate gyrus
	R‐LGN	−54	−22	24	137	5.32	.044	Left supramarginal gyrus
Control < IGE	L‐VLa	44	−12	54	357	5.31	.00011	Right precentral gyrus
		−2	42	−18	103	4.17	.038	Frontal medial cortex
Control > REF‐IGE	R‐VLa	26	−40	−16	230	6.27	.003	Right fusiform gyrus; right cerebellum
	L‐LGN	−40	−44	58	367	5.7	.0001	Left superior parietal lobule
		6	14	26	215	6.45	.0025	Paracingulate gyrus
		38	2	60	127	5.05	.025	Right middle frontal gyrus
	R CM	−8	−14	58	114	5.03	.039	Supplementary motor area
		10	8	44	106	5.15	.039	Anterior cingulate gyrus
Control < REF‐IGE	L‐VLa	46	−12	54	270	5.24	.00068	Right precentral gyrus
Control > nonREF‐IGE	R‐CM	4	48	−18	408	7.98	.00002	Right frontal medial cortex
Control < nonREF‐IGE	L‐MD	46	−56	−14	248	6.72	.0024	Right temporooccipital region
REF‐IGE > nonREF‐IGE	R‐VLa	−12	−70	44	108	5.19	.033	Left lateral occipital gyrus, superior part
	L‐VLp	32	32	56	349	6.17	.000014	Right middle frontal gyrus
	R‐VLp	18	−68	56	271	6.02	.00021	Right lateral occipital gyrus, superior part
		−12	−70	46	159	5.02	.0035	Left lateral occipital gyrus, superior part
		−20	−54	56	116	4.91	.011	Left superior parietal lobule
	R‐CM	12	42	20	100	5.85	.026	Right paracingulate gyrus
REF‐IGE < nonREF‐IGE	R‐CM	58	−22	18	128	5.35	.015	Right parietal operculum cortex

*Note*: k, number of contiguous voxels; Group statistics were presented with absolute T‐score (|T|) and cluster‐size false discovery rate corrected *p*‐value (*p*_*FDR*_).

Patients with non‐refractory IGE had significantly increased functional connectivity between left MD and right temporooccipital cortex and decreased connectivity between right CM and right frontal medial cortex relative to controls (Figure 4b, Table 5). Patients with refractory IGE had significantly increased connectivity between left VLa and right precentral gyrus relative to controls. Compared to controls, patients with refractory IGE had decreased connectivity between right VLa and right fusiform gyrus, between the right CM and supplementary motor area and anterior cingulate gyrus, and between left LGN and left superior parietal lobule, paracingulate gyrus, and right middle frontal gyrus (Figure 4c, Table 5). In direct comparisons between patient groups, patients with refractory IGE had increased functional connectivity between the right VLa and superior part of left lateral occipital gyrus, between the left VLp and right middle frontal gyrus, between the right VLp and bilateral superior part of lateral occipital gyrus, and between the right CM and right paracingulate gyrus. Patients with refractory IGE had decreased functional connectivity between the right CM and right parietal operculum cortex (Figure 4d, Table 5).

## 4 DISCUSSION

There were two primary goals of the present study. First, we sought to determine regional thalamic nuclear volume alterations in patients with refractory and non‐refractory IGE and healthy controls. We report significant volume reduction of the AV nuclei bilaterally only in patients with refractory IGE. Patients with non‐refractory IGE did not show significance or a trend (uncorrected *p* < .05) for volume reduction of these nuclei. Second, we sought to investigate rs‐fMRI thalamocortical connectivity alterations in IGE and to determine whether alterations are associated with treatment outcomes. We report significant alterations in thalamocortical functional connectivity between patients with IGE and controls and found evidence for increased functional connectivity between the VLp nuclei bilaterally and regions of frontal and occipital cortex in patients with refractory IGE relative to patients with non‐refractory IGE.

### 4.1 Biological and clinical implications

VBM studies have not consistently reported atrophy of the anterior regions of the thalamus in IGE, although some have described this in patients with JME (Kim, Kim, Seo, Suh, & Koh, 2013; Mory et al., 2011). On the contrary, one study reported increased volume in the anterior thalamus in patients with IGE relative to controls, which was more obvious in patients with absence seizures, who had worse seizure control (Betting et al., 2006). Our findings that the AV nuclei are specifically affected in patients with refractory IGE is a new finding; the fact that these nuclei are affected in both cerebral hemispheres is consistent with the bihemispheric nature of IGE. The anterior thalamic nuclei complex is a part of the limbic system and an important relay of the Papez circuit (Papez, 1937). The nuclei receive output from the hippocampus via the fornix or mammillary bodies via the mammillothalamic tract, and project to the cingulate cortex; information travels through the cingulate bundle and returns to the hippocampus to complete the circuit (Shah, Jhawar, & Goel, 2012; Weininger et al., 2019). The anterior thalamus has been suggested to relate to multiple cognitive processing tasks such as memory, executive function and spatial navigation (Jankowski et al., 2013; Ketz, Jensen, & O'Reilly, 2015; Wolff & Vann, 2019), which are cognitive domains where patients with IGE are often impaired (Abarrategui, Parejo‐Carbonell, García García, Di Capua, & García‐Morales, 2018; Kim & Ko, 2016; Loughman, Bowden, & D'Souza, 2014; Ratcliffe et al., 2020). Localised anterior thalamic structural alterations may relate to recent work documenting limbic changes in IGE, including hippocampal anatomy, function and metabolism (Caciagli et al., 2019; Lin et al., 2013; Ristić et al., 2011), cingulate diffusion and metabolic changes (Focke et al., 2014; Simani et al., 2020; Sinha et al., 2019; Slinger, Sinke, Braun, & Otte, 2016) and diffusion value alterations of the fornix (Sinha et al., 2019). Our result also fit with those of EEG‐fMRI studies that have demonstrated an association between the anterior thalamic nucleus and generalised SWDs in patients with IGE (Tyvaert et al., 2009). Given the limbic pathways impacted, anterior thalamus deep brain stimulation (DBS) could be considered an attractive target to modulate limbic seizure networks in addition to the modulation of overall thalamocortical excitability (Laxpati, Kasoff, & Gross, 2014). Anterior thalamus DBS is principally used to treat refractory focal epilepsy (Bouwens van der Vlis et al., 2019; Fisher et al., 2010) based on the known limbic involvement in these patients. Despite that, the CM thalamic nucleus is the typical DBS target for refractory IGE (see below), anterior thalamic DBS has been performed in some patients with primary generalised epilepsy (Kim et al., 2017; Krishna et al., 2016; Park et al., 2019). However, it is not clear whether the stimulation of anterior nuclei has any benefit for patients with refractory primary generalised seizures. One study indicated no seizure outcome improvements when stimulating the anterior thalamic and centromedian thalamic nuclei in patients with generalised epilepsy (Alcala‐Zermeno et al., 2021).

Bihemispheric thalamocortical alterations seeded from the ventral lateral nuclei were prominent findings in the present study. We observed significantly increased functional connectivity between these nuclei and cortex in all patients compared to controls (anterior), refractory patient's relative to controls (anterior), and refractory patients relative to non‐refractory patients (anterior and posterior). Collectively this suggests functional hyperconnectivity between ventral lateral nuclei and cortex in patients with refractory generalised seizures. The VLa nucleus receives pallidal afferents and the VLp nucleus receives cerebellar afferents, and projects to the supplementary motor area, premotor cortex and primary motor cortex (Mai & Majtanik, 2019). Hyperconnectivity was observed between these nuclei and primary motor and frontal lobe regions (in addition to superior occipital regions), which is consistent with the known pathophysiology of generalised seizures and hyperactivation and hyperconnectivity during cognitive processing in IGE (Caciagli et al., 2020; Vollmar et al., 2011). Key to our findings is that increased functional connectivity between ventral lateral nuclei and cortex could represent an imaging marker that can help discriminate between refractory and non‐refractory given that such hyperconnectivity was observed in patients with refractory IGE compared to non‐refractory IGE. However, whether this represents a mechanistic marker of pharmacoresistance or is an epiphenomenon of a clinical difference between patient groups (e.g., prevalence of GTCS) remains to be elucidated. To our knowledge, this is the first demonstration of functional nuclei‐cortical connectivity differences between patients with refractory and non‐refractory IGE. Similar to our structural findings, that these findings were bihemispheric is in keeping with the generalised nature of the disorder.

Both patients with refractory IGE and patients with non‐refractory IGE showed decreased functional connectivity between the right CM nucleus and areas of the medial frontal cortex relative to controls. On the basis that the CM‐parafascicular complex contributes to neuromodulation within a basal ganglia and motor network (Mclardy, 1948; Sadikot & Rymar, 2009), the CM nucleus is the DBS target for refractory primary generalised seizures (Valentín et al., 2013; Zangiabadi et al., 2019). CM DBS yields over 50% reduction in seizure frequency across various types of epilepsy and patients with medically refractory generalised epilepsy manifesting primary tonic–clonic or absence seizures benefiting the most (Alcala‐Zermeno et al., 2021; Cukiert et al., 2009; Ilyas, Pizarro, Romeo, Riley, & Pati, 2019; Klinger & Mittal, 2018; Son et al., 2016; Valentín et al., 2013; Velasco et al., 2007; Velasco, Velasco, Jimenez, Velasco, & Marquez, 2002). Our results are in accordance with the rationale for CM DBS as we observed disrupted functional connectivity between the CM nucleus and supplementary motor area in patients with refractory IGE. We did not, however, hypothesise unilateral CM abnormalities given the bilateral electrophysiological nature of IGE. To our knowledge, this is the first demonstration of CM functional connectivity alterations in IGE, which is reassuring, although the unilateral nature of these results was surprising. There is an increasing imaging (Li et al., 2017; Liu et al., 2017; Wang et al., 2011; Zhu et al., 2016) and electrophysiological (Fernandez‐Baca Vaca & Park, 2020) literature reporting unilateral abnormalities in IGE; whether the same is true for the CM requires further studies to support our findings. Moreover, given that, CM connectivity differences were observed between patient groups, and that this region is an effective DBS target for refractory IGE, we suggest that connectivity profiles of this area may additionally have utility as imaging markers of pharmacoresistance. Whereas CM findings may have been expected, we also unexpectedly observed significantly decreased functional connectivity between the left and right LGN and fronto‐parietal cortical regions. The LGN part of the visual pathway (Saalmann & Kastner, 2011; Yuan et al., 2016) and we are not aware of previous work reporting the significance of these nuclei in the generation or modulation of primary generalised seizures. Interconnectivity with the thalamic reticular nucleus may provide insights (Uhlrich, Manning, & Feig, 2003); the thalamic reticular nucleus has been suggested to be involved in generalised SWDs in absence seizures (Huguenard, 2019). A recent study has also proposed that increasing neuronal firing in the thalamic reticular nucleus may enhance the inhibitory activity of the dorsal LGN (Campbell, Govindaiah, Masterson, Bickford, & Guido, 2020).

### 4.2 Methodological considerations

The patient cohort included different subtypes of IGE. Previous studies have suggested that thalamic alterations may be exhibited differently in IGE with different underlying seizure conditions (Betting et al., 2006). We were unable to analyse individual IGE subtypes in this study given the overall sample size; future work should try to replicate our findings separately in patients with refractory and non‐refractory absence epilepsy, juvenile myoclonic epilepsy, and epilepsy with primary generalised tonic–clonic seizures alone. Our primary goal was to prospectively recruit patients according to whether they had refractory or non‐refractory IGE; it is challenging to recruit patients with non‐refractory IGE in particular given that these patients are frequently not in a tertiary care service. Recruiting patients with refractory IGE is less challenging and all our refractory patients were under care at the Walton Centre NHS Foundation Trust at the time of investigation. This accounts for the fewer number of non‐refractory patients recruited. Although we recommend that our findings are replicated in larger groups of patients with non‐refractory IGE, we remain confident of our findings, as our statistical approaches were robust. Furthermore, the refractory IGE group had a higher number of patients with GTCS compared to patients with non‐refractory IGE. Previous work has demonstrated that patients with GTCS are more likely to be medically refractory compared to patients without GTCS (Janmohamed, Brodie, & Kwan, 2020). Therefore, refractoriness may be more closely associated with the seizure disorder and the imaging differences merely an epiphenomenon of the seizure disorder, not necessarily refractoriness. Future work should strive to recruit equal numbers of patients with refractory and non‐refractory IGE and a balanced representation of patients with GTCS.

It is plausible to assume that administration of different ASMs have differential effects on thalamocortical functional connectivity in patients. Previous studies suggested the chronic use of topiramate and carbamazepine—less common ASMs in our cohort—may have effects on BOLD signal measures and negatively impact on cognition in patients with focal epilepsy, although valproic acid, lamotrigine or levetiracetam were unlikely to yields significant effects (Haneef, Levin, & Chiang, 2015; van Veenendaal et al., 2015, 2017). Although there is limited evidence indicating that typically prescribed ASMs used for primary generalised epilepsy affect resting‐state fMRI networks in patients with IGE, it would be prudent to design a study that systematically examines whether this is the case.

Although the probabilistic method of thalamic nuclei segmentation used in the present study corresponds well to a histological atlas of the thalamus (Iglesias et al., 2018), the approach does not delineate nuclear subfields and is inherently constrained by the limited spatial resolution and contrast of T1w MRI. Optimising sequences to improve tissue contrast (Iglesias et al., 2018; Lee, Seo, & Park, 2020; Su et al., 2019) and high‐field delineation of thalamic regions (Kanowski et al., 2014; Liu, D'Haese, Newton, & Dawant, 2020; Plantinga et al., 2014) may increase the reliability of segmentations. The accuracy of nuclei segmentations and limited spatial resolution may also impact rs‐fMRI connectivity; the time series from adjacent nuclear regions may impinge on some of the observed thalamocortical functional correlations. Furthermore, we have suggested that regional thalamic structural and functional alterations shown in our study may be relevant for cognitive impairments previously observed in patients with IGE. However, we urge caution with this interpretation given that we did not collect cognitive data on our participants and therefore we were unable to investigate this potential correlation directly. Finally, it is important to be mindful that interictal epileptiform discharges may impact on regional glucose metabolism, rs‐fMRI data and resultant functional networks in patients with IGE (Aghakhani et al., 2015; Cheng, Yan, Xu, Zhou, & Chen, 2020; Dahal et al., 2019; Gotman et al., 2005; Kim, Im, Kim, Lee, & Kang, 2005; Lv, Wang, Cui, Ma, & Meng, 2013). We did not perform simultaneous EEG‐fMRI to control for interictal epileptiform discharges in this study and this will be an important for future resting‐state functional network studies in IGE. Moreover, our study would also have been strengthened by the inclusion of ambulatory EEG or video EEG data. Ambulatory EEG data is not routinely collected in patients with IGE in the United Kingdom. Finally, given that, the LGN is one of the smallest nuclei, our method of probabilistic segmentation may have overestimated the LGN region of interest and functional correlations may have been confounded by adjacent nuclei. Further work is needed to determine whether functional connectivity alterations of the LGN are due to the disorder or methodological confounds.

## 5 CONCLUSION

Atrophy of the anterior thalamic nuclei and resting‐state functional hyperconnectivity between ventral lateral nuclei and cerebral cortex may be imaging markers that help discriminate between refractory and non‐refractory patients with IGE. These structural and functional abnormalities fit well with the known importance of thalamocortical systems in the generation and maintenance of primary generalised seizures, and the increasing recognition of the importance of limbic pathways in IGE.

## CONFLICT OF INTEREST

None of the authors has any conflict of interest to disclose.

## Data Availability

The data that support the findings of this study are available on request from the corresponding author. The original data are not publicly available due to ethical restrictions.
